# Health-Related Quality of Life and Frequency of Depressive Episodes Among Healthcare Professionals in an Outpatient Health Facility in Italy: A Comparison Between 2017 (Pre-COVID) and 2025 (Post-COVID)

**DOI:** 10.3390/jcm15020874

**Published:** 2026-01-21

**Authors:** Antonio Urban, Michela Atzeni, Giulia Cossu, Massimo Tusconi, Cesar Ivan Aviles Gonzales, Gabriele Finco, Clelia Madeddu, Laura Atzori, Caterina Ferreli, Elisabetta Cotti, Mauro Carzedda, Stefano Lorrai, Maria Cristina Deidda, Alessandra Bertolino, Pedro José Fragoso Castilla, Shellsyn Giraldo Jaramillo, Fernanda Velluzzi, Roberta Montisci, Elisa Cantone, Enzo Tramontano, Fabrizio Bert, Viviana Forte, Marcello Nonnis, Mauro Giovanni Carta

**Affiliations:** 1 PhD Program in Capacities Building for Global Health, University of Cagliari, 09124 Cagliari, Italy; 2 Obesity Unit, Department of Medical Sciences and Public Health, University of Cagliari, 09124 Cagliari, Italy; 3 Department of Medical Sciences and Public Health, University of Cagliari, 09124 Cagliari, Italy; 4 Department of Medicine and Surgery, University of Kore, 94100 Enna, Italy; 5 Dermatology Clinic, Department of Medical Sciences and Public Health, University of Cagliari, 09124 Cagliari, Italy; 6 Department of Surgical Sciences, Division of Dentistry, University of Cagliari, 09124 Cagliari, Italy; 7 Center for Palliative Care and Pain Management, University Hospital of Cagliari, 09124 Cagliari, Italy; 8 PhD Program in Tropical Medicine, Universidad Popular del Cesar, Valledupar 200004, Colombia; 9 Microbiology Program, Universidad Popular del Cesar, Valledupar 200004, Colombia; 10 Faculty of Health, Universidad Popular del Cesar, Valledupar 200004, Colombia; 11 Department of Life and Environmental Sciences, University of Cagliari, 09124 Cagliari, Italy; 12 Department of Public Health and Pediatrics Sciences, University of Torino, 10124 Torino, Italy; 13 Department of Pedagogy, Psychology, Philosophy, University of Cagliari, 09124 Cagliari, Italy; 14 Department of Nursing, Universidad Popular del Cesar, Valledupar 200004, Colombia

**Keywords:** quality of life, COVID-19, health professionals, depressive symptoms

## Abstract

**Background/Objectives**: The COVID-19 pandemic severely impacted healthcare systems globally, with Italian healthcare professionals experiencing heightened stress, organizational challenges, and a significant psychological burden. This study investigates the frequency of depressive symptoms and health-related quality of life (H-QoL) among outpatient healthcare workers in Italy, comparing pre-pandemic (2017) and post-pandemic (2025) periods. **Methods**: A cross-sectional study was conducted in 2025, including 97 healthcare professionals from five outpatient departments at the University Hospital of Cagliari. Participants completed demographic surveys, the Short Form Health Survey (SF-12), and the Patient Health Questionnaire (PHQ-9) to assess H-QoL and depressive symptoms. Data were compared with previously published data from the same facility collected in 2017 and with pre-pandemic Italian community surveys. **Results**: Compared to 2017, there was a statistically significant increase in depressive episodes (38.1% vs. 33.2%, *p* = 0.01) and a higher proportion of individuals with low H-QoL (62.9% vs. 43.5%, *p* < 0.0001) in 2025. After age- and sex-standardization, both depressive symptoms and low H-QoL were significantly more prevalent among healthcare professionals in 2025 compared with the general population before the pandemic. Within the 2025 sample, non-medical healthcare workers showed a significantly higher prevalence of depressive symptoms than medical doctors, while female healthcare workers were more likely to report low H-QoL. **Conclusions**: Despite the pandemic’s end, healthcare workers, especially those in outpatient settings, continue to face elevated psychological distress. Specific professional and gender-related vulnerabilities persist, and structural challenges, such as staff shortages and organizational issues, may exacerbate this burden. Sustained mental health support and targeted systemic interventions remain crucial to mitigate the long-term impact on the healthcare workforce.

## 1. Introduction

The COVID-19 pandemic posed a major challenge to the healthcare systems worldwide [1,2,3,4], exposing or exacerbating organizational issues and generating significant stress among healthcare workers [5,6], including in Italy [7].

The impact of the pandemic on healthcare personnel was particularly severe in Italy. Firstly, the country was among the hardest hit during the initial phases of the pandemic, being the first Western nation to be affected, with an initially very high mortality rate even among healthcare professionals [8,9]. Secondly, the widespread belief, even among health workers, that the Italian healthcare system was one of the best in the world and would withstand the crisis without major issues, was contradicted by the emergence of clear organizational unpreparedness and lack of resources. This led to feelings of powerlessness and frustration among healthcare professionals [2,4,10,11].

Furthermore, during the pandemic, satisfaction levels among healthcare professionals working outpatient services were significantly lower than those reported by mental health professionals [12]. This result was interpreted as a consequence of the crisis affecting the healthcare system during COVID-19. The evaluated outpatient services were located in hospital settings [13]. The “siege-like” conditions during COVID, in which professionals had to limit interpersonal contact for fear of infecting family and friends, likely placed these professionals in a more difficult position compared to those working in mental health care [14], which in Italy is typically provided through community-based services [12]. While several studies have documented the acute psychological impact of the pandemic on healthcare workers, less evidence is available on the persistence of depressive symptoms and reduced quality of life in the post-pandemic period, particularly in outpatient hospital settings.

The main objective of this study is to assess whether the situation has improved after the pandemic by comparing the frequency of depressive symptoms and the prevalence of low perceived quality of life in the outpatient healthcare professionals sample assessed in 2025, with results obtained from a previously published investigation conducted in the same facility in 2017 (i.e., pre-pandemic) [15]. In addition, the present findings are compared with results from two community surveys conducted in the Italian general population before the COVID-19 pandemic [16,17].

## 2. Materials and Methods

### 2.1. Study Design

We conducted a cross-sectional study in 2025 and performed a diachronic comparison with a healthcare worker sample assessed in 2017, as well as with pre-pandemic community samples.

### 2.2. Study Sample

A voluntary sample of healthcare workers from five outpatient units (pain therapy, oncology, dermatology, endocrinology, cardiology) at a University Hospital in Sardinia, Italy, was recruited. Data collection was conducted in October 2025, during the same week across all participating units, after approval from the ethics committee and with the cooperation and consent of the unit directors. Data were collected over three morning shifts and two afternoon shifts. Participants were included in the study if they fulfilled the following eligibility criteria: (i) being at least 18 years of age; (ii) belonging to either sex; (iii) having provided written informed consent; and (iv) being actively employed in a healthcare role. Eligible professional profiles comprised physicians, nurses, psychologists, healthcare assistants (OSS), biologists, social workers, rehabilitation professionals, educators, as well as postgraduate trainees enrolled in psychology or medical specialties. Only one doctor and one nurse declined participation because they were too busy and reported not having time available (2.06% of the overall contacted sample). In total, 97 health professionals participated in the study and were included in the current study sample. All questionnaires were fully completed and included in the analysis; no cases were excluded due to missing data.

### 2.3. Study Tools

After having signed a declaration of informed consent (approved by the ethics committee), health professionals were asked to complete the following:

(1) A questionnaire on general work and demographic data (age, gender, place of employment and occupational role) already used in the cited previous survey [15]. To preserve anonymity, as the crossing of the data could have allowed identification, less frequent professions (e.g., social worker, nutritionist, dentistry or agent of security, or) were grouped into a broad category.
(2) The Short Form Health Survey in the 12 items version (SF-12) [18,19] in its Italian validated version [20]. The SF-12 measures of the Health-Related Quality of Life (H-QoL) inquiring various aspects this construct, including emotional status, social functioning, general health status, pain, vitality, and mental health. The total score (range of 12–47) is obtained by summing the Likert-scale score of each item with higher scores indicating a higher level of H-QoL. We adopted a cut-off of 36/37 according to previous survey for detecting a low level of H-QoL computing a score of less than 1 standard deviation from the average of an Italian community sample [16].
(3) The 9 items Patient Health Questionnaire (PHQ-9) [21], a self-administered tool, well-established as a tool for screening depressive symptoms and assessing their severity. Each item refers to a core symptom relevant to the diagnosis of depressive episode according to the Diagnostic and Statistical Manual of Mental Disorders (DSM-5) [22]. The total score of this tool is the sum of the item scores. Each item is rated on a 4-point Likert scale from 0 (“Not at all”) to 3 (“Nearly every day”), with higher scores indicating greater severity of depressive symptoms. The total score is obtained by summing the item ratings, with scores of 4–5 indicating at least mild depressive symptoms and scores of 9–10 indicating symptoms of at least moderate severity [23]. The scale demonstrates good internal reliability, with a Cronbach’s α of 0.89 [23]. For this study, the validated Italian version of the instrument was used [24,25]. To identify clinically relevant depressive symptomatology in this study, we adopted a cut-off of ≥8 on the PHQ-9, consistent with a previous study on healthcare professionals [12]. Participants scoring ≥ 8 were classified as positive for depressive symptoms.

### 2.4. Description of the 2017 Population and Study Protocol

For diachronic comparison, data from a previous cross-sectional survey conducted in 2017 in the same outpatient health facility were used as the pre-pandemic reference population [15]. The 2017 study included 510 healthcare professionals (physicians, nurses, and administrative/technical staff). Socio-demographic characteristics were collected, including age and sex.

The 2017 survey used the same psychometric instruments used in the present investigation, namely:

- the Patient Health Questionnaire-9 (PHQ-9) to screen for depressive symptoms, using the validated Italian version and a cut-off score of ≥8;
- the Short Form Health Survey-12 (SF-12) to assess Health-Related Quality of Life (H-QoL), with low QoL defined as scores below one standard deviation from the Italian normative sample.

Further methodological details of the 2017 study, including sampling procedures and data collection methods, are reported in the original publication [15].

### 2.5. Statistical Analysis

The sample size was determined based on the previous study involving healthcare workers, which used a similar methodology and the PHQ9 measurement tool [15]. Given that the sample consisted of approximately 510 subjects who had shown a frequency of PHQ9 > 8 of 33%, 81 or more measurements/surveys were needed to have a 95% confidence level that the true value was within ±10% of the measured/surveyed value. This approach ensured that the study had sufficient statistical power to detect significant effects or associations between the main variable of interest, with the sample size based on the size used in the previous study on the same population of healthcare workers before the pandemic.

A diachronic comparison was performed between the proportion of healthcare workers screened positive on the PHQ-9 in the 2025 sample and those identified in the 2017 survey conducted in the same outpatient facility before the COVID-19 pandemic [15]. The same cut-off of 8 was applied, consistent with the methodology of the previous study. The comparison was carried out by means of chi square test, the limit of confidence of Odds Ratio were calculated by means of Miettinen’s simplified method.

To compare the 2025 healthcare worker sample with the two normative community datasets collected prior to the pandemic [16,17], direct age- and sex-standardization was performed to account for demographic differences between samples.

Similarly, the prevalence of low Health-Related Quality of Life (H-QoL) in the 2025 sample was compared with the normative community datasets [16,17] through direct standardization. For detecting a low level of H-QoL we adopted a cut-off of 36/37 according to previous survey, computing a score of less than 1 standard deviation from the average of the Italian community sample [19]. A two-tailed *p*-value < 0.05 was considered statistically significant. The statistical analyses were conducted using Stata software, version 17.0.

## 3. Results

Table 1 shows the socio-demographic characteristics, including gender (male prevalence) and age (individuals older than 49 years), of healthcare professionals assessed in 2025 compared with those evaluated in 2017 [15], as well as with two normative community samples collected prior to the COVID-19 pandemic, used to compare the frequency of people with low H-Qol [16] and the frequency of PHQ-9 positives [17].

The two healthcare worker samples (2017 vs. 2025) were well-balanced in terms of age, gender and professional distribution (medical doctors vs. non-medical doctors). The 2025 healthcare worker sample showed a lower frequency of people aged > 49 compared to the normative sample 1 (PHQ-9) [17], although the difference did not reach the statistical significance (37.1% vs. 44.8%; *p* = 0.141). A similar difference was observed when compared with normative sample 2 (SF-12) [16] (37.1% vs. 42.2%; *p* = 0.297).

Conversely, the proportion of men was significantly lower in the 2025 healthcare worker sample compared with both normative samples: 29.9% vs. 48.5% in normative sample 1 (*p* < 0.001) and 29.9% vs. 43.1% in normative sample 2 (*p* = 0.010).

Table 2 summarizes the prevalence of PHQ-9–positive depressive symptoms (PHQ-9 ≥ 8) and low Health-Related Quality of Life (SF-12 ≤ 36) among healthcare professionals assessed in 2025, compared with the pre-pandemic healthcare worker sample from 2017 [15], as well as with pre-pandemic normative community samples [16,17].

In the comparison between healthcare professionals assessed in 2025 and those evaluated in 2017, the prevalence of depressive symptoms was higher in the post-COVID sample (38.1% vs. 33.2%; *p* = 0.010). Similarly, the proportion of individuals reporting low H-QoL was significantly higher in 2025 compared with 2017 (62.9% vs. 43.5%; *p* < 0.001).

When comparing the 2025 healthcare worker sample with the general population reference datasets collected prior to the COVID-19 pandemic [16,17], direct age- and sex-standardization was applied to account for demographic differences between samples. After standardization, the prevalence of PHQ-9–positive cases among healthcare professionals was markedly higher than in the normative sample (40.2% vs. 13.1%; *p* < 0.001). Likewise, the standardized prevalence of low H-QoL was substantially higher in the 2025 healthcare worker sample compared with the normative population (58.8% vs. 23.7%; *p* < 0.001).

If we consider the association of different parameters (age, gender, and professional role) with depressive symptoms and low Health-Related Quality of Life within the 2025 healthcare worker sample (Table 3), no significant associations emerged between gender or age and the presence of depressive symptoms. Conversely, non-medical healthcare workers showed a significantly higher prevalence of depressive symptoms compared with medical doctors (46.8% vs. 22.6%; χ^2^ = 5.424; *p* = 0.020). With regard to Health-Related Quality of Life, female healthcare workers were significantly more likely to report low H-QoL compared with their male counterparts (70.6% vs. 44.8%; χ^2^ = 5.780; *p* = 0.016; OR = 2.95; 95% CI 1.2–7.2).

## 4. Discussion

The present study reveals a persistently high prevalence of depressive symptoms and low Health-Related Quality of Life (H-QoL) among outpatient healthcare workers in Italy, even in the post-pandemic context of 2025. Compared to data collected in 2017, before the onset of COVID-19, there has been a statistically significant increase in both the frequency of depressive symptoms and low quality of life. These results are consistent with findings from previous studies conducted during the pandemic [8,12,26], and with some preliminary reports in the post-pandemic [27], which suggest that the psychological impact of COVID-19 on healthcare workers has had long-lasting consequences [28,29,30,31,32,33,34,35,36]. Our results confirm that even after five years after the onset of the pandemic, its effects on healthcare workers continued to be detected, similarly to what was found in other reports, although with a shorter interval of time [34,35]. This confirms what has been highlighted following other epidemics [36], suggesting that large-scale health crises may produce long-term psychological consequences on healthcare personnel, particularly when structural vulnerabilities are already present.

One of the key findings is that, despite the formal end of the pandemic emergency, the mental health burden among healthcare professionals remains alarmingly high. In particular, the elevated rates of depressive symptoms and poor quality of life highlight that recovery has not occurred uniformly across all professional domains. These findings may reflect unresolved systemic issues, such as chronic staff shortages, poor organizational support, and the cumulative stress experienced by healthcare workers over the past few years, as consistently reported in recent international reviews on healthcare workforce well-being [37].

Interestingly, our analysis shows that non-medical staff (such as nurses, technicians, and other support roles) are significantly more likely to experience depressive symptoms compared to medical doctors. This disparity may be explained by differences in levels of autonomy, responsibility, professional recognition, and work-related stress exposure [38,39]. Furthermore, salaries for nursing and healthcare technicians in Italy are among the lowest in Europe. The average gross annual salary of an Italian nurse is lower than the European average, and even after adjusting for cost of living, Italian nurses earn less than their counterparts in many other European countries [40]. Economic strain has been identified as an additional risk factor contributing to psychological distress among non-medical healthcare workers, particularly in post-pandemic contexts [41]. It is therefore possible that the post-COVID state of unease caused by the pandemic’s impact on healthcare workers could also be amplified by economic problems.

Female healthcare workers were more likely to report a low quality of life, which aligns with the literature suggesting that women in healthcare often face additional burdens, including emotional labor, work–life imbalance, and gender-based discrimination [6,7,42].

Another relevant comparison was made between the 2025 sample normative data collected in the general population prior to the pandemic [16,43]. The analysis confirms that outpatient healthcare professionals report significantly worse mood state and H-QoL than the general population in pre-COVID era. This finding may reflect a persistent psychological burden in this professional group, potentially indicating a heightened vulnerability to pandemic-related stressors.

The differences between outpatient healthcare workers and those employed in community-based mental health services, as reported in previous studies [12], may be explained by the specific nature of hospital work during the pandemic. Hospital-based outpatient professionals experienced more direct exposure to COVID-19 patients, stricter isolation protocols, and greater workload, often without the psychological support mechanisms available in community mental health care.

The elevated stress levels found in this study (i.e., high risk of depression and of low quality of life) among the staff, could also be related to the fact that several sources have revealed that the Italian national health system is going through (particularly in the southern regions) a serious organizational crisis and also related to the scarcity of resources with consequent lack of staff and lowering of the quality of the services provided [44].

Despite the waning of the pandemic emergency, this study underscores the need for ongoing psychological monitoring and support for healthcare personnel, especially those working in outpatient hospital settings. Interventions should be tailored not only to support individuals already affected by psychological distress but also to promote preventive strategies and structural changes within the healthcare system. From an organizational perspective, potential strategies may include interventions aimed at improving working conditions, such as adequate staffing levels, clearer role definitions, and increased involvement of non-medical staff in clinical and organizational decision-making processes [4,10]. In addition, structured burnout prevention programs, access to confidential psychological support services, and measures addressing work–life balance may be particularly relevant for this professional group [26,29,32]. While the present study was not designed to evaluate the effectiveness of specific interventions, these findings underline the importance of adopting differentiated and profession-sensitive approaches when planning mental health support strategies for healthcare workers.

### Limitations

This study has some limitations. First, it relies on self-reported data, which may be affected by response bias. Second, the sample was recruited from a single hospital in Sardinia and may not fully represent the broader Italian healthcare workforce. Lastly, the cross-sectional design prevents the establishment of causal relationships. However, the use of consistent tools across time points strengthens the reliability of longitudinal comparisons.

## 5. Conclusions

In conclusion, the mental health and perceived quality of life of outpatient healthcare professionals remain significantly impaired in the post-pandemic period. These findings stress the importance of sustained psychological support and systemic reforms to address the persistent vulnerabilities within the healthcare workforce.

## Figures and Tables

**Table 1:** Socio-demographic characteristics of healthcare workers (2025 vs. 2017) and comparison with normative community samples [15,16,17].

	Health Workers 2025 N (%)	Health Workers 2017 * N (%)	Test (χ^2^), *p*; OR (95% CI) (2025 vs. 2017)	Normative Sample 1 ** N (%)	Test (χ^2^), *p*; OR (95% CI) (2025 vs. Norm1)	Normative Sample 2 *** N (%)	Test (χ^2^), *p*; OR (95% CI) (2025 vs. Norm2)
Men gender	29 (29.9)	173 (33.9)	χ^2^ = 0.595 *p* = 0.441 OR = 0.83 (0.5–1.3)	729 (48.5)	χ^2^ = 12.126 *p* < 0.001 OR = 0.46 (0.3–0.7)	1000 (43.1%)	χ^2^ = 6.642 *p* = 0.010 OR = 0.56 (0.4–0.9)
Age > 49	36 (37.1)	176 (33.8)	χ^2^ = 0.243 *p* = 0.622 OR = 1.12 (0.7–1.7)	673 (44.8)	χ^2^ = 2.169 *p* = 0.141 OR = 0.73 (0.5–1.1)	985 (42.2%)	χ^2^ = 1.190 *p* = 0.297 OR = 0.80 (0.5–1.2)
Medical Doctors	35 (36.1)	192 (36.8)	χ^2^ = 0.235 *p* = 0.638 OR = 0.89 (0.6–1.4)	Not Applicable		Not Applicable	
TOT	97	510		1503		2320	

* Pre-pandemic healthcare worker sample. Carta et al., 2017 [15]. ** Normative community sample 1 (PHQ-9). Moro et al. 2015 [17]. *** Normative community sample 2 (SF-12). Carta et al. 2012 [16].

**Table 2:** Depressive Episodes and Low H-Qol among healthcare professionals (2025 vs. 2017) and comparison with normative community samples.

	Health Workers 2025 N (%)	Health Workers 2017 * N (%)	Test (χ^2^), *p*; OR (95% CI) (2025 vs. 2017)	Normative Samples ** N (%)	Test (χ^2^), *p*; OR (95% CI) (2025 vs. Norm)
Score PHQ-9 ≥ 8	37 (38.1)	173 (33.2)	χ^2^ = 6.049 *p* = 0.01 OR = 1.86 (1.1–3.0)	-	-
Score PHQ-9 ≥ 8 (standardized)	39 (40.2)	-	-	197 (13.1)	χ^2^ = 53.215 *p* < 0.001 OR = 4.46 (2.9–6.9)
Score SF-12 ≤ 36	61 (62.9)	222 (43.5)	χ^2^ = 12.700 *p* < 0.001 OR = 2.21 (1.4–3.4)	-	-
Score SF-12 ≤ 36 (standardized)	57 (58.8)	-	-	551 (23.7)	χ^2^ = 50.625 *p* < 0.001 OR = 4.57 (3.0–6.9)

* Pre-pandemic healthcare worker sample. Carta et al., 2017 [15]. ** Normative community samples: Moro et al., 2015 (PHQ-9) [17] and Carta et al., 2012 (SF-12) [16]. Comparisons with normative samples were performed after direct age- and sex-standardization [16,17].

**Table 3:** Associations between depressive symptoms (PHQ-9 ≥ 8), low health-related quality of life (SF-12 ≤ 36), and socio-demographic variables in the 2025 healthcare worker sample.

	Variable	Category	N/Total (%)	Test (χ^2^), *p*; OR (95% CI)
PHQ-9 ≥ 8	Gender	Women	26/68 (38.2)	χ^2^ = 0.001 *p* = 0.977 OR (W) = 1.01 (0.4–2.5)
		Men	11/29 (37.9)	
	Age	>49 years	13/36 (36.11)	χ^2^ = 0.100 *p* = 0.751 OR (young) = 1.14 (0.5–2.7)
		<50 years	24/61 (39.3)	
	Profession	Medical doctors	8/35 (22.6)	χ^2^ = 5.424 *p* = 0.020 OR (N-MD) = 2.97 (1.2–7.5)
		Non-medical doctors	29/62 (46.8)	
SF-12 ≤ 36	Gender	Women	48/68 (70.6)	χ^2^ = 5.780 *p* = 0.016 OR (W) = 2.95 (1.2–7.2)
		Men	13/29 (44.8)	
	Age	>49 years	20/36 (55.5)	χ^2^ = 1.318 *p* = 0.251 OR (young) = 1.64 (0.7–3.8)
		<50 years	41/61 (67.2)	
	Profession	Medical doctors	18/35 (51.4)	χ^2^ = 3.080 *p* = 0.079 OR (N-MD) = 2.14 (0.9–5.0)
		Non-medical doctors	43/62 (69.2)	

OR (W) = women vs. men; OR (young) < 50 vs. >49; OR (N-MD) = non-medical vs. medical doctors.

## Data Availability

The datasets of this study will not be publicly available due to individual privacy rules.
